# Incorporation of Porcine Adenovirus 4 Fiber Protein Enhances Infectivity of Adenovirus Vector on Dendritic Cells: Implications for Immune-Mediated Cancer Therapy

**DOI:** 10.1371/journal.pone.0125851

**Published:** 2015-05-01

**Authors:** Ivy Wilkinson-Ryan, Julius Kim, Sojung Kim, Ferhat Ak, Lindzy Dodson, Marco Colonna, Matthew Powell, David Mutch, Dirk Spitzer, Ted Hansen, Simon P. Goedegebuure, David Curiel, William Hawkins

**Affiliations:** 1 Washington University School of Medicine, Division of Gynecologic Oncology, St. Louis, United States of America; 2 University of Chicago, Department of Neuro-oncology, Chicago, United States of America; 3 Washington University School of Medicine, Department of Pathology and Immunology, St. Louis, United States of America; 4 Saint Jans Gathuis Hospital, Department of SJG Apotheek, Weert, The Netherlands; 5 Saint Louis University, Department of Biology, St. Louis, United States of America; 6 Washington University School of Medicine, Division of Surgery, St. Louis, United States of America; 7 Washington University School of Medicine, Division of Cancer Biology, St. Louis, United States of America; Baylor College of Medicine, UNITED STATES

## Abstract

One strategy in cancer immunotherapy is to capitalize on the key immunoregulatory and antigen presenting capabilities of dendritic cells (DCs). This approach is dependent on efficient delivery of tumor specific antigens to DCs, which subsequently induce an anti-tumor T-cell mediated immune response. Human adenovirus serotype 5 (HAdV5) has been used in human studies for gene delivery, but has limited infection in DCs, which lack the proper receptors. Addition of the porcine fiber knob (PK) from porcine adenovirus type 4 to HAdV5 allows the virus to deliver genetic material via binding to glycosylated surface proteins and bypasses the coxsackie-and-adenovirus receptor required by wild-type HAdV5. In this study we explored the potential therapeutic applications of an adenovirus with PK-based tropism against cancers expressing mesothelin. Infectivity and gene transfer assays were used to compare Ad5-PK to wild-type HAdV5. Mouse models were used to demonstrate peptide specificity and T-cell responses. We show that the PK modification highly augmented infection of DCs, including the CD141+ DC subset, a key subset for activation of naïve CD8+ T-cells. We also show that Ad5-PK increases DC infectivity and tumor specific antigen expression. Finally, vaccination of mice with the Ad5-PK vector resulted in enhanced T-cell-mediated interferon gamma (IFN-γ) release in response to both mesothelin peptide and a tumor line expressing mesothelin. Ad5-PK is a promising tool for cancer immunotherapy as it improves infectivity, gene transfer, protein expression, and subsequent T-cell activation in DCs compared to wild-type HAdV5 viruses.

## Introduction

Dendritic cells (DCs) can be engineered to yield a clinical benefit when an antigen is delivered directly to DCs for presentation and immune stimulation [1–4]. For instance, DCs loaded with tumor associated antigens can prime and activate T-cells to mount an anti-tumor immune response. One potential pitfall of DC mediated immunotherapy is inadequate antigen loading as well as antigen expression by regulatory DC subsets, which can lead to immune tolerance [5]. Antigen delivery systems must result in high levels of expression by the proper DC subsets to avoid tolerance. Recent studies have shown that the CD141^+^ (or BDCA3^+^) DC subset in humans, which is equivalent to the CD8α^+^ subset in mice, is the most efficient at cross-presenting antigens on MHC I to naïve CD8^+^ T-cells, which is essential for cytotoxic T lymphocytes (CTL)—mediated immune responses to viruses and tumor antigens [2,4,6]. Practical exploitation of DCs involves extra-corporeal antigen delivery as a peptide, or genetically via vector-mediated infection [1,7,8]. Currently, DC manipulation is under investigation for a variety of cancers and represents a promising vehicle for cancer immunotherapy [1–4,7,9,10].

Adenoviruses such as human adenovirus serotype 5 (HAdV5) are commonly used for gene and immunotherapy because of their ability to deliver genetic material to non-dividing cells such as DCs [11–15]. The clinical application of HAdV5 is limited by the relative paucity of its receptor, the coxsackie-and-adenovirus receptor (CAR), on dendritic cells [11–13]. We and others have shown that the CAR binding domain of HAdV5 can be replaced with knobs of different serotypes allowing for CAR-independent binding as well as receptor targeting [14,15]. The porcine knob mediates adenoviral infection through glycosylated cell surface proteins which are expressed by DCs and is CAR independent [16–19]. Preliminary studies of Ad5PK have shown that incorporation of the fiber knob does not alter the viral growth kinetics, thermostability, or increase clearance *in vivo* [18]. In this paper, we explore the incorporation of the fiber knob from porcine adenovirus type 4 (PAdV4).

The ability of DCs to induce an anti tumor immune response is dependent both on the method of antigen delivery to the DC and the properties of the antigen itself [3]. Mesothelin is a membrane-bound glycoprotein expressed by mesothelial cells in the lung pleura, pericardium, and peritoneum of healthy individuals and overexpressed in pancreatic and epithelial ovarian cancers making it a potential immune target [20]-[21,22]. Mesothelin is essential to invasion and metastasis of cancer cells and the side effects of destroying normal mesothelin producing cells is minimal [23–25]. *In vivo* murine and early phase human trials using mesothelin-targeted therapies have shown mesothelin specific CTLs and improved survival [9,26,27]. In this study we used a mesothelin peptide incorporated into a single chain trimer (SCT) construct encoding peptide and HLA-2.1 to overcome issues of processing and presentation associated with tumor antigens [28,29].

The goal of this study was to explore the potential applications of *ex vivo* DC antigen loading by Ad5-PK for the treatment of mesothelin expressing cancers. By including the mesothelin SCT into the Ad5 and Ad5-PK constructs, we were able to compare expression of a clinically meaningful tumor antigen on DCs and the subsequent T-cell response. This work is significant because the ability to infect immunostimulatory DCs and enhance expression of target antigens are important steps towards effective cancer immunotherapy.

## Materials and Methods

### Cell Lines

Human embryonic kidney (HEK) 293 and SKOV3 cell lines were purchased from the American Type Culture Collection (ATCC; Manassas, VA), cultured in media containing 10% fetal bovine serum, (FBS; Hyclone; Logan, UT), 2mM L-glutamine, 100 U/ml penicillin, and 100 mg/ml streptomycin (Mediatech, Inc., Herndon, VA). SKOV3-HLA-A2 (SKOV3A2) cells were kindly gifted from Dr. Timothy Eberlein.

To generate murine DCs, bone marrow from C57BL/6 or HHDII mice was harvested from tibias and femurs. Red blood cells (RBC) were lysed with RBC lysis buffer (Sigma Aldrich, Saint Louis, MO). Cells were plated in 6 well plates at 1-3×10^5^ cells/ml in complete medium containing RPMI 1640, 10% fetal calf serum, 1% glutamax, 1% kanamycin sulfate, 1% sodium pyruvate, and 1% nonessential amino acids (all from Life Technologies, Grand Island, NY). Medium was supplemented with murine GM-CSF (Miltenyi, Auburn, CA) and cells were cultured for 6 days to obtain immature dendritic cells. This study was approved and carried out in accordance with Washington University School of Medicine Division of Comparative Medicine guidelines for care and use of laboratory animals (protocol 20130036).

To generate human DCs, peripheral blood mononuclear cells (PBMCs) were isolated from leukocyte reduction filters from anonymous healthy platelet donors. PBMCs were cultured in RPMI with 2mM glutamine (Gibco, Grand Island NY), 1% non-essential amino acids, 1% HEPES buffer (Mediatech Inc, Manassas, VA), 2% pooled human AB serum, 100 U/ml penicillin (Gibco), and 100μg/mL streptomycin (Gibco). After 2 hours non-adherent cells were collected and cryopreserved. Adherent cells were cultured in DC media with 100ng/mL GM-CSF (Miltenyi, Auburn, CA) and 20ng/mL IL-4 (Miltenyi). GM-CSF and IL-4 was replaced every 3 days. All cells were incubated at 37°C in 5% CO_2_ in humidified conditions. The Washington University School of Medicine Human Research Protection Office Review Board (protocol 201108117) approved this protocol. No consent was required by the Human Research Protection Office Review Board for anonymous healthy donors.

### Plasmid construction

Mesothelin containing plasmid pMH103 was gifted by Dr. Ira Pastan [30]. Full length mesothelin (FLM) was amplified from pMH103 by using following primers: Meso-F-HindIII: 5’- ACTTAAGCTTtgccaggctctccACCCCA-3’ and Meso-R-EcoRV: 5’-ATCTGATATCTCAGGCCAGGGTGGAGGCT-3.’ Amplified mesothelin and pShuttle CMV were digested with HindIII and EcoRV. Following gel purification, double digested mesothelin was ligated into linearized pShuttle-CMV, generating pShuttle-CMV-Meso

A nine amino acid mesothelin peptide (VLPLTVAEV, 9mer) has previously been shown to be immunogenic and HLA-A2-restricted according to the BIMAS database [31,32]. MHC Class-I restricted peptides are only capable of activating CD8^+^ T cells thus enabling the use of unselected lymphocytes for activation detection assays such as IFN-γ ELISpot. The SCT construct including a mammaglobin peptide (pING.PADRE-mamA2.1dtSCT) has been previously described and was gifted to our lab from Drs. Ted Hansen and William Gillanders [33]. The nucleotide sequences for mamA2.1 was excised with NheI and AgeI, and replaced with the nucleotide sequences corresponding to 9mer (5’-CCGGTTTGTAT GCTGTGCTGCCGCTGACCGTGGCGGAAGTGGGATGCGGTG-3’, purchased from IDT, Coralville, IA), generating pING.PADRE-9merstSCT. pShutte-CMV-9SCT was generated by ligating XbaI digested pShuttle-CMV and pING.PADRE-9merstSCT. Sequencing analysis was performed at each step to ensure integrity of the construct.

### Generation of recombinant adenovirus

The recombinant plasmid DNA of Ad5-9SCT (or Meso, GFP1, or Luc1) was generated by homologous recombination in E.coli BJ5183 with PmeI-linearized pShuttle CMV-9SCT (or Meso, GFP1, or Luc-1) and Ad5 backbone [34]. After confirming correct recombination by sequencing analysis, the viruses were rescued and propagated in HEK293 cells. Virus was purified by two rounds of CsCl gradient ultracentrifugation. Viral particle (VP) concentration was determined at 260 nm by using a conversion factor of 1.1x10^12^ viral particles per absorbance unit [35]. The Ad5PK-9SCT (or GFP1 or Luc1) was generated in the same way except pVK500-PK which contains the Ad5 backbone underwent replacement of the Ad5 knob for the Porcine knob as a backbone, as previously described [16]. Construction of Ad5/3Luc1 and Ad5Luc.FF/CD40L has been previously described [36–38]. Viruses with different backbones and plasmids were constructed separately and represent different batches of virus.

### Virus infection and gene transfer assay

**Fig 1:**
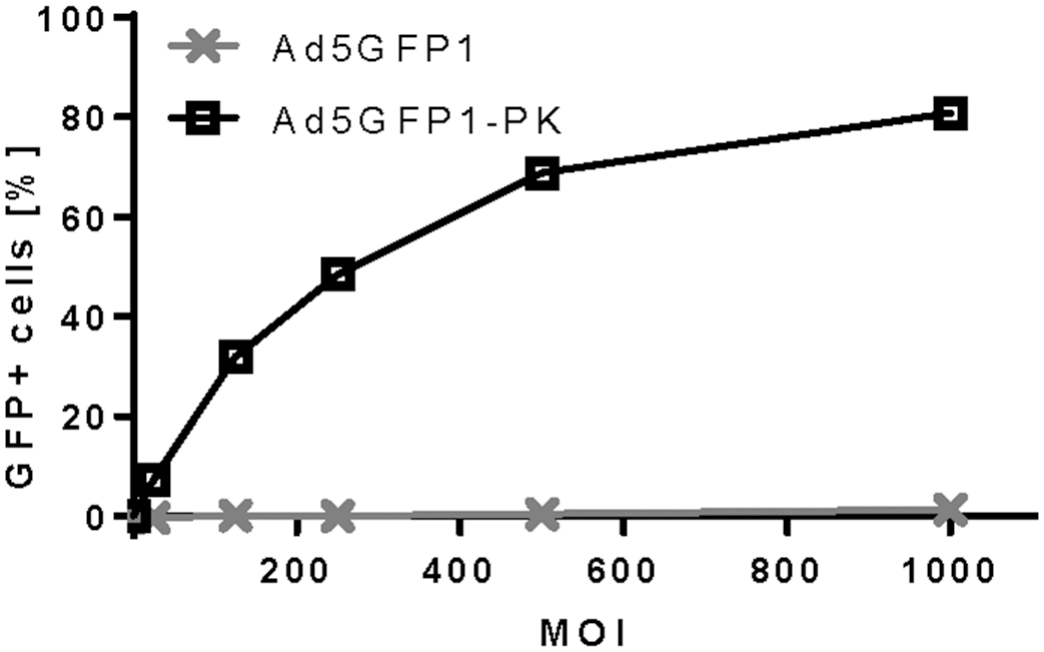
Titration of Ad5-GFP1 and Ad5-GFP1-PK. Human DCs were infected with Ad5-GFP1 or Ad5-GFP1-PK at 0, 25, 125, 250, 500, and 1000 MOI. After 24 hours, the percent GFP-positive DCs was determined by FACS analysis to establish the MOI range for comparative assays. Cells with DC morphology based on forward and side scatter plots were isolated. Ad5-GFP1-PK showed higher GFP expression at every MOI value, with MOI ≤500 within the linear range of gene expression.

DCs were incubated overnight in 24 well plates with 3-5×10^5^ cells/well. Three hundred to 500 VP/cell were used for infection based on viral titration assays (Fig 1). After 2 hours of coincubation with the virus, cells were washed, and incubated for 24–48 hours prior to analysis. For gene transfer assays, cells were harvested in passive lysis buffer (Promega, Madison, WI) and luciferase activity was determined following the manufacturer’s instruction (Promega). Luciferase activity is reported as relative light units (RLU). To assess rates of viral infection and gene expression, DCs were infected with Ad5-GFP1 or Ad5-GFP1-PK and analyzed via FACS as described below. Luciferase assays in conjunction with GFP FACS assays demonstrates the number of cells infected, the rate of gene transfer, and the rate of gene expression. For inhibition of *O*-linked glycosylation, DCs were incubated with α-benzyl-*O*-GalNAc (1μg/ml) (Sigma-Aldrich) overnight before infection.

### FACS analysis

Virus infected or uninfected (control) cell lines were blocked with Human TruStain FcX (Biolegend, San Diego, CA). For PBMC staining and CD141 detection, anti-human CD141 (Biolegend), anti-human CD11c (Biolegend), and anti-human lineage cocktail 1 (Lin1) (Biolegend) were used. For SCT detection DCs were stained with anti-human HLA-A2 (BD Pharmigen) antibody. Mouse DCs were identified using anti-mouse CD11c antibody (Biolegend). All FACS data was acquired on FACSCalibur (BD Biosciences, San Jose, CA) and analyzed with FlowJo software (FlowJo, Ashland, OR).

### Mouse models and T-cell assays

HHDII transgenic (B6; Cg-B2M^tm1Unc^ H2-D1^tm1Bpe^Tg (HLA-A2/H2-D/B2M)1Bpe) mice that express the transgene, Tg (HLA-A2/H2-D/B2M) 1Bpe, in a mixed background involving B2M^tm1Unc/tm1Unc^ and H2-D1^tm1Bpe/tm1Bpe^[39] were gifted by Dr. F. Lemonnier. These mice are designated HHDII. For peptide specificity assays, eight week old HHDII mice were primed with 5X10^10^ viral particles (VP) intramuscularly with either Ad5-9SCT or Ad5-9SCT-PK. Mice were boosted *in vivo* with 1X10^10^ VP/mouse two weeks after priming and splenocytes were harvested nine days after boost injections. For the *ex vivo* boost experiments, all mice were primed with intramuscular Ad5-Meso (an adenovirus expressing full length mesothelin) and harvested nine days after priming. Prior studies have shown that murine intramuscular vaccination with adenovirus leads to migration of infected DCs to local lymph nodes followed by activation and dissemination of T-cells [40,41]. Mouse splenocytes were boosted *ex vivo* with a 1:10 ratio of DCs infected with Ad5-9SCT or Ad5-9SCT-PK (500 MOI) to splenocytes for five days in the presence of 50 units/mL of murine IL-2 (Miltenyi Auburn, CA). ELISpot was performed per kit protocol (Mabtech, Mariemont, OH). After blocking the ELISpot plate, splenocytes were plated at 2.5X10^5^ cells/well. For peptide specificity studies 20μg/mL of the relevant (9mer) or irrelevant peptide were used. The irrelevant peptide is a ten amino acid sequence (KLLGPHVEGL) from a different portion of the mesothelin protein, but with high HLA-A2 binding. For tumor studies, 5X10^4^ SKOV3 wild-type (SKOV3wt) control cells or SKOV3A2 cells were plated in each well and incubated overnight at 37°C. Spots were detected with R4-6A2-biotin antibody, Streptavidin-ALP, and BCIP/NBT substrate per kit protocol. Each cell combination was run in triplicate. ELISpot plates were analyzed on CTL-ImmunoSpot S6 Micro Analyzer (CTL, Shaker Heights, OH).

### Statistical Method

All assays were run in triplicate and the results are expressed as a mean of the triplicate and standard deviation. Assays were carried out at least twice to verify results. FACS plots shown in the figures are representative, with the triplicate data combined and expressed graphically. Data generated with human dendritic cells was tested in at least two donors. GraphPad Prism 6 (La Jolla, CA) was used to generate graphs and perform statistical analysis. P-values were generated using Student’s t-test.

## Results

### Ad5-PK increases infection efficiency over tropism modified Ad5 on iDCs

**Fig 2:**
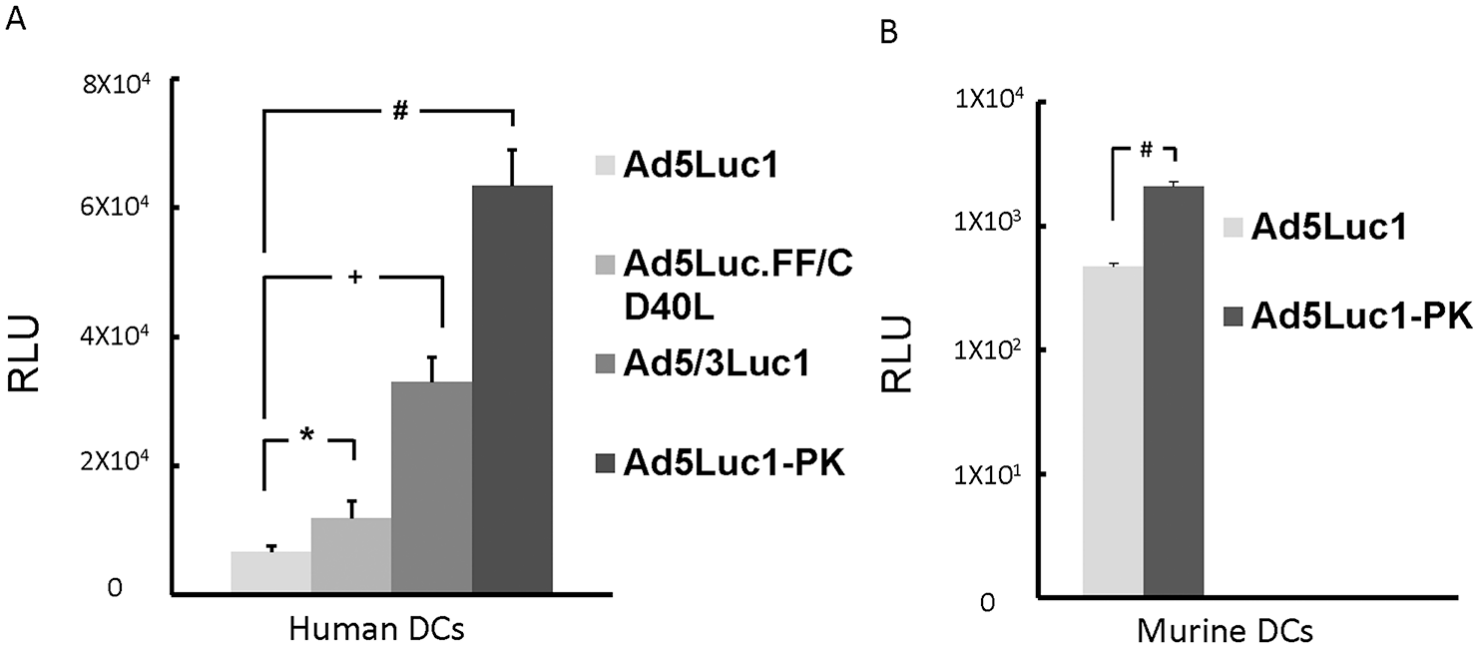
A. Ad5PK enhances DC gene transfer compared to previously reported Ad5 constructs. Human immature DCs were infected with Ad5Luc1, Ad5Luc.FF/CD40L, Ad5/3Luc1 or Ad5Luc1-PK at an MOI of 300 VP/cell. Luciferase activity (relative light unit, RLU) was determined in 48hr post infection. All fiber modified Ad5 vectors showed infectivity enhancement in iDC. *, +, and # indicate p = 0.02, p = 0.0005 and p<0.0001, respectively. B. Murine iDC were plated in triplicate and infected with Ad5Luc1-PK and Ad5Luc1 (control) at an MOI of 300 VP/cell. Luciferase activities were measured after 48 hrs. Ad5Luc1-PK showed a 4-fold increase in murine iDCs of gene transfer activity (“#” indicates p<.003).

To investigate infection efficiency of Ad5-PK in immature dendritic cells (iDCs), the gene transfer assay was performed with a panel of genetically modified adenoviruses that are currently undergoing study for clinical application: Ad5Luc1 (wild type), DC infectivity enhanced Ad5/3Luc1, DC targeted Ad5Luc.FF/CD40L, and Ad5Luc1-PK. All viral vectors contain a luciferase reporter gene under the CMV promoter. As expected, all modified Ad5 vectors showed statistically significant infectivity enhancement of human iDCs over wild type Ad5 (Fig 2A, p<.003 for all comparisons). Infection efficiency of glyco-targeted Ad5Luc1-PK was superior to that of Ad5/3Luc1. Based on the significant increase in gene infection observed with Ad5-PK, this viral vector was selected for further analysis.

In order to establish the efficacy of Ad5-PK in animal models, we evaluated gene transfer efficiency in mouse DCs via luciferase expression. Ad5Luc1-PK shows superior infection efficiency over Ad5Luc1 in murine iDCs (mean 472 v. 2087) (Fig 2B, p<0.003).

### Ad5-PK increases infection frequency of iDCs

**Fig 3:**
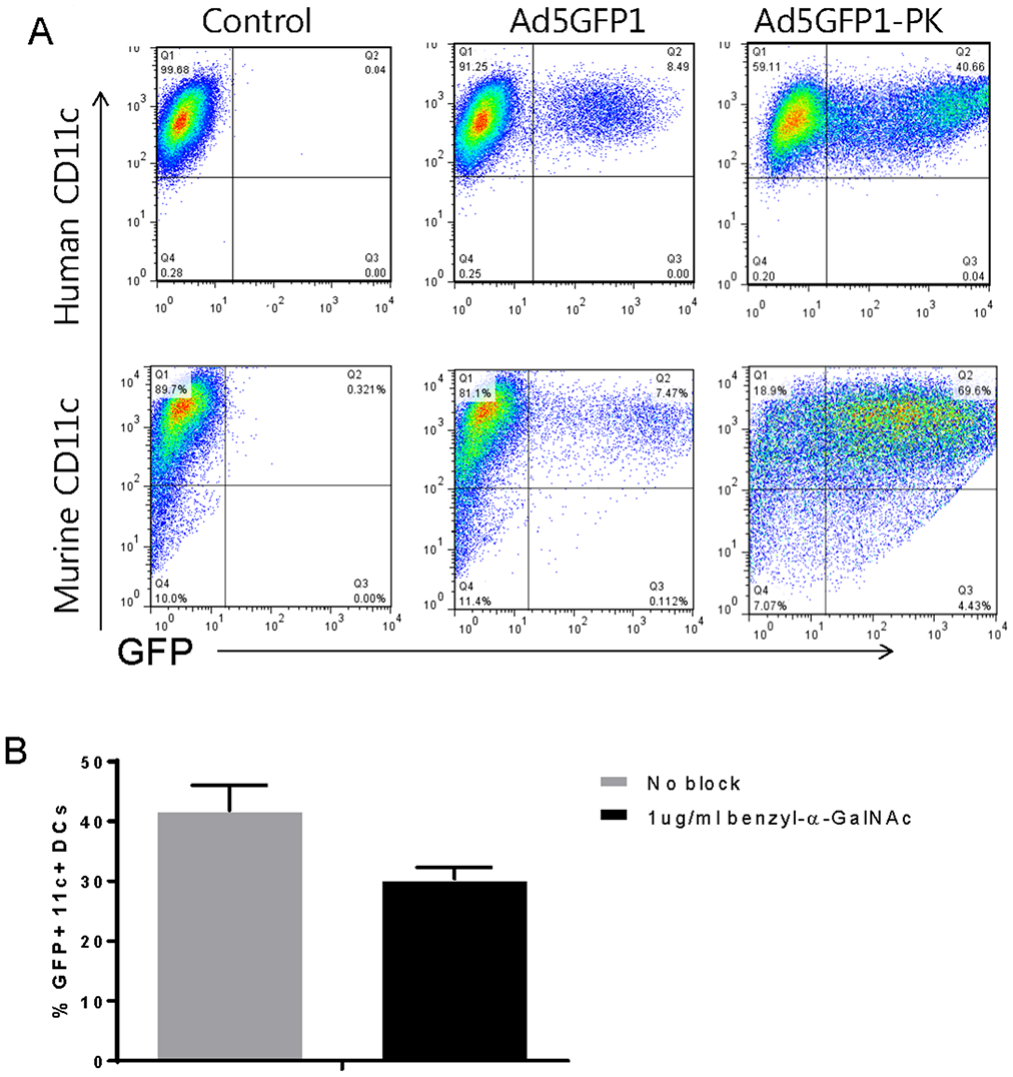
Ad5GFP1-PK infection induces higher levels of GFP in iDC compared to wild-type Ad5GFP1 (A) Human and mouse iDCs were infected with 500 MOI Ad5GFP1-PK or Ad5GFP1. After 24 hrs, FACS analysis was performed to determine GFP positive cells in CD11c^+^ DCs. In both human and murine DCs, infection with Ad5GFP1-PK lead to a higher percentage of GFP-expressing DCs than infection with Ad5GFP1. (B) O-linked glycoprotein synthesis was blocked overnight with benzy-α-benzyl-GaINAc. After infection with Ad5GFP1-PK, the expression of GFP in DCs was reduced by 25% (p = .02).

To determine the percentage iDCs infected by virus, Ad5 and Ad5-PK encoding enhanced GFP (GFP1) were generated [16]. Ad5GFP1-PK was analyzed for its ability to infect human and mouse iDCs compared to Ad5GFP1. Infection of Ad5GFP1-PK resulted in an almost 5-fold enhancement of the efficiency of human iDCs and an over 9-fold enhancement in murine iDCs compared to Ad5GFP1 (Fig 3A). This assay was performed in four different human donors with a range of 3.4–5.7-fold increase in GFP expression with the Ad5GFP1-PK vector. Data from a single human donor and a single mouse are shown in fig 3A. It has previously been shown that the knob domain on fibers of Ad5-PK preferentially utilizes cell surface *O*-linked glycoproteins for cell entry [16]. The infectivity of Ad5GFP1-PK decreased 25% after treating cells with α-benzyl-*O*-GalNAc (1μg/ml), an inhibitor of *O*-linked glycosylation (Fig 3B, p = 0.02). Ad5-PK shows enhanced infectivity and efficient gene transfer in human iDCs, partially through a binding preference for *O*-linked surface glycoproteins. In combination with our previous results, this confirms that Ad5-PK vectors have superior gene transfer ability and infectivity compared to wild type Ad5.

### Ad5-PK demonstrates increased infection of DC subsets specialized in activation of naïve CTL

**Fig 4:**
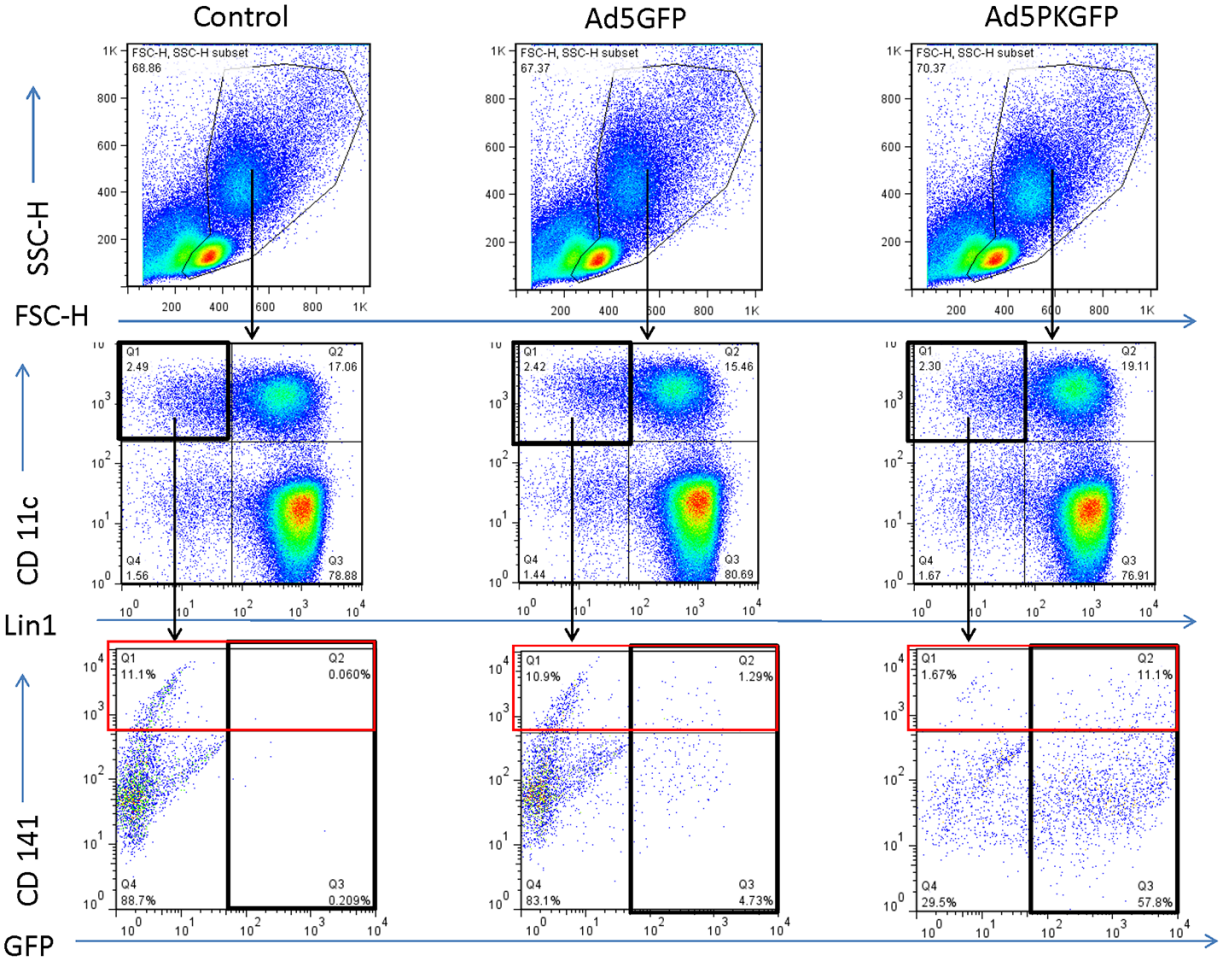
Ad5GFP-PK infects both CD141^+^ and CD141^-^ DCs. Human PBMCs were infected with either Ad5GFP1 or Ad5GFP1-PK and analyzed by flow cytometry for expression of GFP in CD141^+^ and CD141^-^ DCs after 24 hours. The DC population was identified by first selecting the CD11c^+^/Lin1^-^ population of live PBMCs (top and middle panel). CD141^+^ DCs were then selected from the CD11c^+^/Lin1^-^ cohort and are outlined in red in the gating schema (bottom panel). All CD11c^+^/Lin1^-^ cells that express GFP are outlined in black in the bottom panel. There was no significant difference in percent DCs or percent CD141^+^ cells between groups (p = .15).

**Fig 5:**
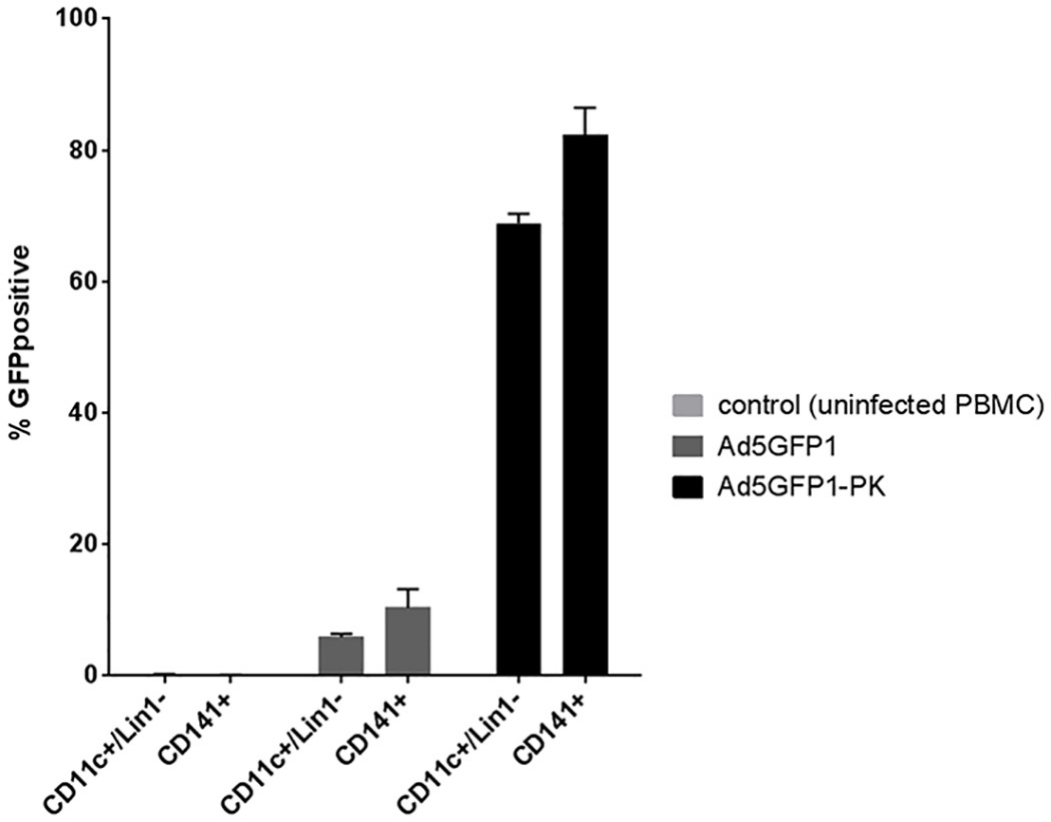
Bar graph showing percent GFP in various DC subsets based on the data from Fig 4. Ad5GFP1-PK infected about 8-times more DCs (including the CD141^+^ subset of DCs) than Ad5GFP1 (p<.0001). This is consistent with our results of cultured DCs.

Recent studies have demonstrated the superior ability of CD141^+^ dendritic cells to cross prime naïve CD8^+^ T cells [42,43]. In order to assess the infection frequency among this subpopulation, PBMCs were infected with either Ad5GFP1 or Ad5GFP1-PK. Uninfected PBMCs were used as negative control. Twenty-four hours after infection cells were stained with CD11c, Lin1, and CD141 and assessed via FACS analysis for expression of GFP. There was no difference in percent of CD141^+^ DCs between groups prior to infection (p = .15, Fig 4). After 24 hours, 69% of all DCs infected with Ad5GFP1-PK were GFP positive and 82% of the CD141^+^ population expressed GFP. This was significantly more than the 10% of CD141^+^ cells infected with Ad5GFP1 (Fig 5, p<.0001). Consistent with other DC infections, only 6% of all DCs infected with Ad5GFP1 expressed GFP. These results show that the infectivity of Ad5-PK is more effective than that of Ad5 at infecting the subpopulation of DCs, best suited for CTL activation.

### DC infection with Ad5-PK leads to increased expression of tumor specific antigens

**Fig 6:**
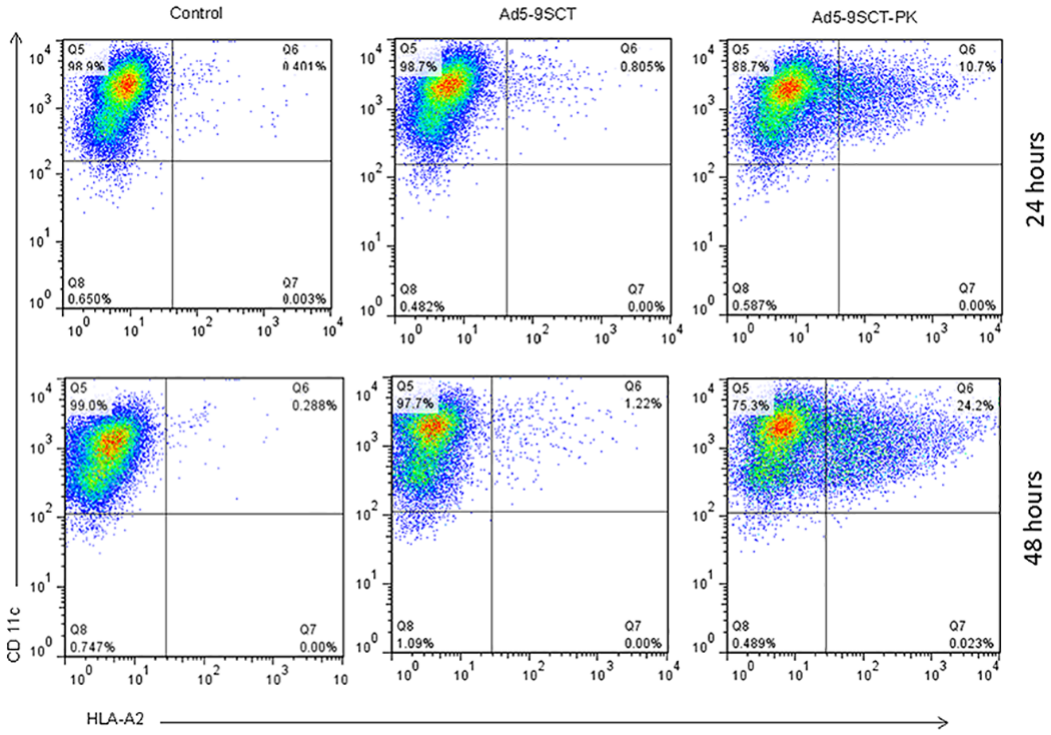
Ad5-9SCT-PK induces efficient expression of antigen in DCs. iDCs were infected at 500 MOI with Ad5-9SCT, or Ad5-9SCT-PK and analyzed for expression of cell surface expression of HLA-A2 (SCT) via FACS analysis after 24 and 48 hours.

**Fig 7:**
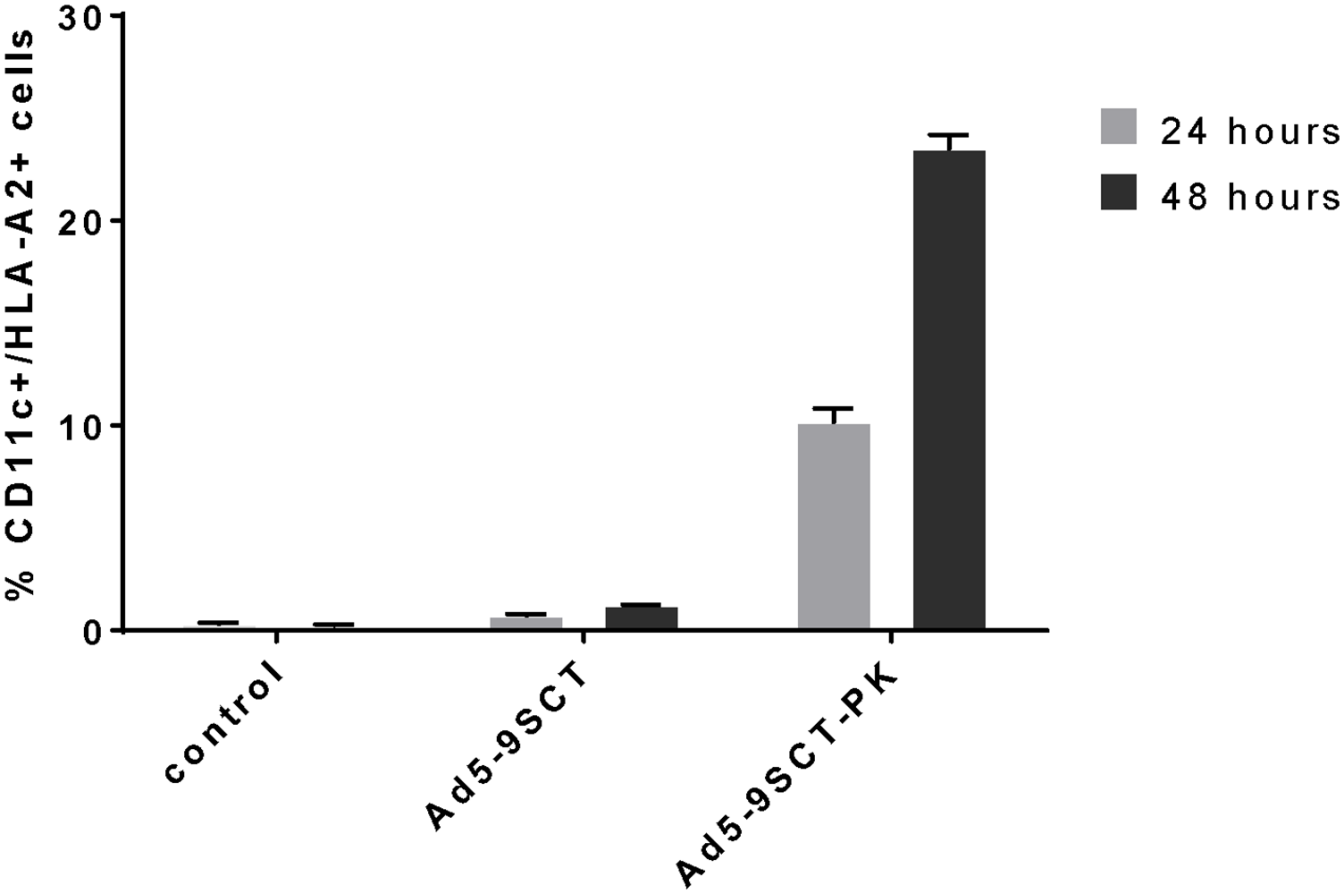
iDCs infected with Ad5-9SCT-PK had a higher expression of the tumor related mesothelin peptide (9mer) SCT at 24 (15-fold increase) and 48 hours (20-fold increase) than cells infected with Ad5-9SCT.

The Ad5-PK has not been assessed for delivery of clinically relevant tumor antigens to DCs. We investigated the potential application of Ad5-PK for *ex vivo* iDC antigen loading for pancreatic and ovarian cancer immune therapy. The HLA-A2.1-binding mesothelin peptide, p530–538 was incorporated into the HLA-A2 single chain trimer construct (9SCT) and encoded in the E1 region of Ad5-PK under the CMV promoter to generate Ad5-9SCT-PK. Human iDCs negative for HLA-A2 were infected with Ad5-9SCT-PK or Ad5-9SCT and analyzed via FACS analysis after 24 and 48 hours for expression of cell surface HLA-A2. Ad5-9SCT-PK infected DCs showed superior expression of HLA-A2 compared to Ad5-9SCT infected DCs (Fig 6, p<.0001). After 24 hours there was a 15-fold increase in HLA-A2 expression on Ad5-9SCT-PK infected DCs compared to Ad5-9SCT infected DCs and after 48 hours the expression improved to an almost 20-fold increase in HLA-A2 expression (Fig 7). Thus, the enhanced infectivity and transduction efficiency seen with Ad5-PK compared to Ad5 leads to increased expression of clinically relevant tumor antigens in DCs.

### Ad5-PK vaccination leads to T-cell specificity and enhances response to tumor cells expressing mesothelin

**Fig 8:**
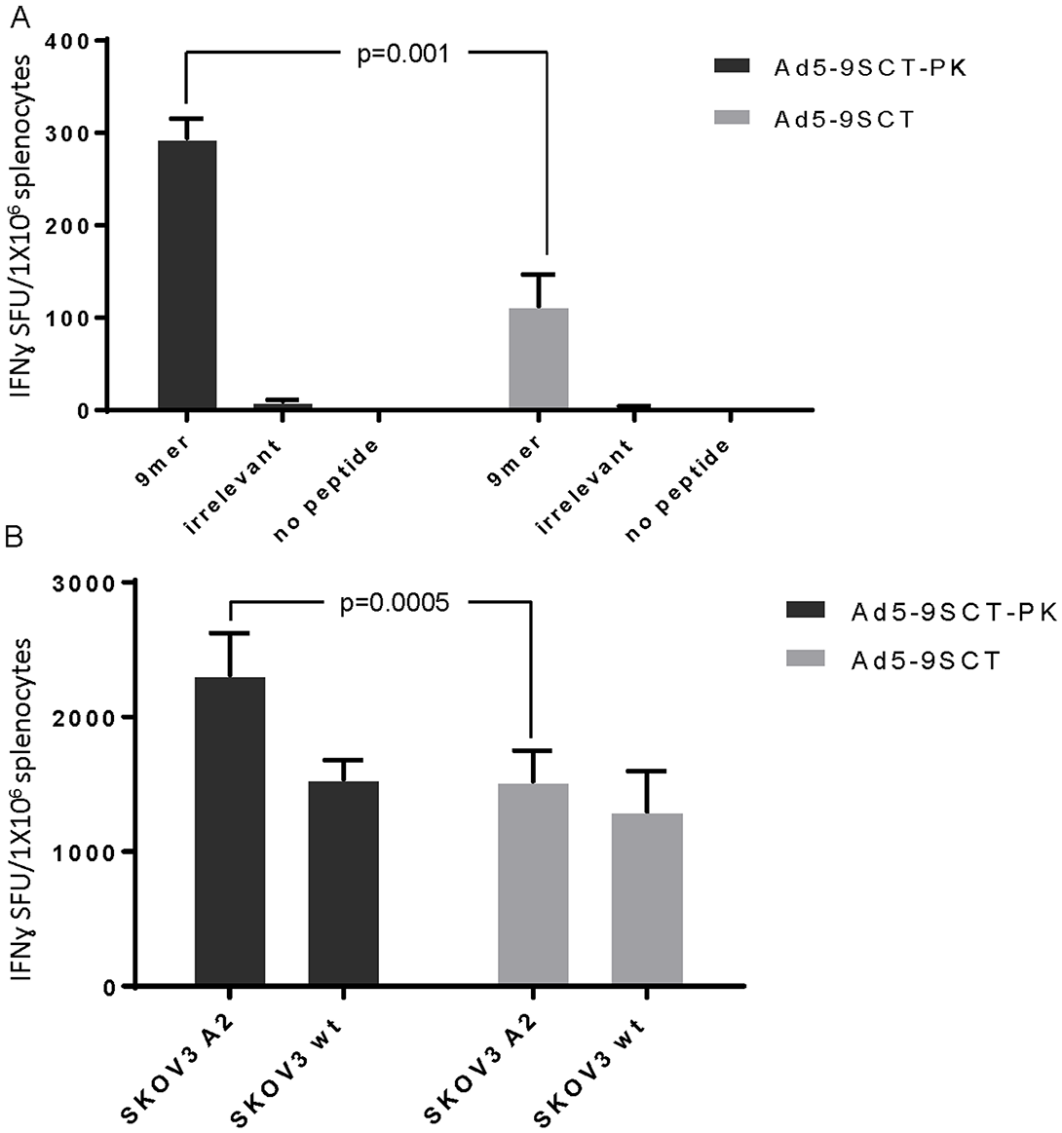
Ad5-9SCT-PK confers specific T cell reactivity. A. Splenocytes from mice vaccinated with Ad5-9SCT or Ad5-9SCT-PK were tested for reactivity against the SCT-encoded 9mer peptide, an irrelevant peptide, or no peptide. Splenocytes from mice injected with Ad5-9SCT-PK had a two-fold increase in the number of IFN-γ-producing cells compared to splenocytes from mice injected with Ad5-9SCT (p = .001) based on ELISpot. B. Mice were primed *in vivo* with HAd5V-containing full length mesothelin. Splenocytes were boosted *ex vivo* with either Ad5-9SCT or Ad5-9SCT-PK infected DCs. Cells boosted with Ad5-9SCT-PK had a 1.5-fold greater IFN-γ response to HLA-A2^+^, mesothelin expressing ovarian cancer cells than Ad5-9SCT (p = .0005).

In order for vaccination to be effective, infected DCs must elicit a T-cell response against cells expressing the relevant antigen (mesothelin 9mer in this case). HHDII mice expressing the transgene, HLA-A2 were vaccinated with either Ad5-9SCT or Ad5-9SCT-PK, and were sacrificed 9 days following boost injections. Splenocytes were isolated and analyzed for antigen specificity using an IFN-γ ELISpot assay. Both A5-9SCT and Ad5-9SCT-PK induced a peptide-specific T cell response. However, mice vaccinated with Ad5-9SCT-PK had twice the response to the 9mer peptide than mice injected with Ad5-9SCT (Fig 8A, p = .001).

To model *ex vivo* vaccination, mice exposed to mesothelin through vaccination with HAdV5 containing full length mesothelin were sacrificed and splenocytes were boosted with Ad5-9SCT or Ad5-9SCT-PK infected murine DCs *in vitro*. Splenocytes were subsequently tested for recognition of the mesothelin-expressing ovarian cancer cell line, SKOV3 and SKOV3 expressing HLA-A2 using an IFNγ ELISpot assay. Splenocytes stimulated with Ad5-9SCT-PK infected DCs contained more HLA-A2-restricted, mesothelin specific T cells than splenocytes stimulated with Ad5-9SCT infected DCs. This was evidenced by a significantly higher frequency of IFN-γ producing cells in response to SKOV3-A2 compared to SKOV3. (Fig 8B, p<0.05). Based on our T-cell experiments, the enhanced infectivity and gene transfer rate of Ad5-PK in DCs leads to more peptide specific T-cells and an increased anti-tumor T-cell response compared to T-cells sensitized or boosted with wild-type HAdV5.

## Discussion

The tumor microenvironment is well adapted to escape immune surveillance. Effective antigen presentation remains one of the many challenges to generating cancer immune therapies. Failure to present the antigen of interest in adequate concentrations by immune stimulatory DCs can lead to tolerance instead of CTL activation. Ad5-PK is a vehicle that can potentially improve anti-cancer CTL activation by increasing the expression of the protein of interest on DCs. We showed that Ad5-PK infects CD141^+^ DCs, a key DC subset relevant to activation of naïve T-cells. We also observed that tumor specific antigen loading via Ad5-PK into iDCs induced superior antigen-specific T-cell responses. Our data indicate that Ad5-PK is a promising therapeutic gene delivery system that may help overcome the hurdles of DC antigen loading and DC-based cancer therapy.

Several strategies have been used to enhance the utility of adenoviruses in cancer therapy by circumventing the CAR receptor. Pereboev *et al* showed increased gene expression in DCs as well as DC activation and enhanced T-cell specificity using CD40 ligand targeted adenovirus in mouse models [44]. CD40 targeted viruses have subsequently been used in pre-clinical studies for the treatment of prostate and cervix cancer as well as melanoma [10,45,46]. A chimeric virus of HAdV5 with the human adenovirus serotype 3 fiber knob (Ad5/3) increases gene uptake in dendritic cells, ovarian cancer, and malignant glioma cells via binding to CD80 and CD86 [36,47,48]. Intraperitoneal administration of Ad5/3 vaccine was well tolerated in a recent phase I trial in ovarian cancer patients [49]. Tumor antigen loading onto DCs via Ad5/3 has not yet been studied in pre-clinical models. Finally, HAdV5 with the fiber knob from canine adenovirus serotype 1 increases infectivity in CAR deficient cells, but has not yet been tested on dendritic cells [50]. In this study, replacement of the HAdV5 fiber knob with the fiber knob from PAdV4 enhanced gene transfer and gene expression compared with CD40 targeted and Ad5/3 adenoviruses.

Ad5-PK enables highly efficient tumor antigen loading in DCs, but we do not believe it is suitable for *in vivo* use in its current form. In order to adapt Ad5PK for *in vivo* use alterations to decrease infectivity of cells that are not professional antigen presenting cells and additional strategies increase immune stimulation will likely be necessary. The necessary modifications are dependent on the mode of delivery of the virus. Prior animal and human studies have used intramuscular, intraperitoneal, mucosal, subcutaneous, and intravascular delivery systems for adenovirus vaccination. If delivered systemically, HAdV5 can cause liver toxicity via its natural liver tropism. Blocking the interaction between coagulation factor X (FX) and hexon proteins of HAdV5 can minimize the liver tropism. Exchange of the hexon gene of Ad5-PK to Adenovirus serotype 3, which has shown lower FX binding affinity, will decrease liver tropism and may reduce vector-related toxicities. It is likely that Ad5-PK targets cell populations other than DCs *in vivo*. DC specific promoters (e.g.CD11c) can control antigen expression at a transcriptional level thus limiting gene expression to DCs. *In vivo* use of wild-type and modified HAdV5 has thus far had an acceptable toxicity profile; testing of the PK modified adenovirus in an immunocompetent animal model will be necessary to fully elucidate side effects and toxicities prior to *in vivo* use in humans [14, 49]. Additional benefit may be gained with use of immune modulating adjuvants such as anti-PDL-1, anti-CTLA-4, GM-CSF, or IL-2 to overcome tolerance and optimize anti-tumor immunity. Vectors using murine mesothelin in an immune competent mouse model with a native ovarian cancer are under development and will allow *in vivo* testing of the vaccine with these immune modulators.

Adenoviral cancer therapy requires overcoming both cancer-induced immune inhibition, and innate immune regulation systems to mount a CTL response to cancer antigens. The PK tropism leads to increased DC antigen loading, which is necessary to avoid tolerance and drive T-cell activation. Ad5-PK is a promising advancement in tumor antigen delivery systems that in addition to viral targeting and adjuvant immune therapy may improve efficacy of immune based cancer therapies.
